# Fabrication and Characterization of New Functional Graded Material Based on Ti, Ta, and Zr by Powder Metallurgy Method

**DOI:** 10.3390/ma14216609

**Published:** 2021-11-02

**Authors:** Izabela Matuła, Grzegorz Dercz, Maciej Sowa, Adrian Barylski, Piotr Duda

**Affiliations:** 1 Institute of Materials Engineering, University of Silesia in Katowice, 41-500 Chorzów, Poland; adrian.barylski@us.edu.pl; 2 Faculty of Chemistry, Silesian University of Technology, 44-100 Gliwice, Poland; maciej.sowa@polsl.pl; 3 Faculty of Science and Technology, Institute of Biomedical Engineering, University of Silesia in Katowice, 41-200 Sosnowiec, Poland; piotr.duda@us.edu.pl

**Keywords:** titanium-based alloy, porosity, FGM

## Abstract

In view of the aging population and various diseases worldwide, the demand for implants has been rapidly increasing. Despite the efforts of doctors, engineers, and medical companies, the fabrication of and procedures associated with implants have not yet been perfected. Therefore, a high percentage of premature implantations has been observed. The main problem with metal implants is the mechanical mismatch between human bone and the implant material. Zirconium/titanium-based materials with graded porosity and composition were prepared by powder metallurgy. The whole samples are comprised of three zones, with a radial gradient in the phase composition, microstructure, and pore structure. The samples were prepared by a three-step powder metallurgy method. The microstructure and properties were observed to change gradually with the distance from the center of the sample. The x-ray diffraction analysis and microstructure observation confirmed the formation of diffusive connections between the particular areas. Additionally, the mechanical properties of the obtained materials were checked, with respect to the distance from the center of the sample. An analysis of the corrosion properties of the obtained materials was also carried out.

## 1. Introduction

Medical reports have presented alarming statistics on the development of diseases and the demand for implants worldwide. The most common long-term bone implant procedure is hip alloplasty [1]. The National Joint Registry registered 790,000 such procedures between 2003 and 2015 in England [2]. It has been estimated that, by the end of 2030, the number of total hip replacements will increase by 174%, compared to that in 2005 [3]. Despite the many efforts of engineers and medical companies, the implantation procedures and materials have not yet been perfected. Therefore, a high percentage of early re-implantations has been observed; for example, in England, considering more than 800,000 procedures, it was necessary to perform almost 90,000 hip endoprosthesis re-implantations [2].

At present, titanium-based alloys in particular, Ti-6Al-4V, are widely used for bone implants; however, such alloys still have a number of disadvantages that need to be improved. The most serious defects of titanium alloys for orthopedic surgery include their high Young’s modulus compared to human bone, low shear strength, and low abrasion resistance. Additionally, there have been reports in the literature regarding the alloying additives such as Al and V, which can be harmful to the human body and, thus, pose a threat to the health and life of patients [4].

The first method is to indirectly affect the mechanical properties by chemical composition control. For example, L.Verestiuc et al. [5,6] analyzed the effect of Si addition on Ti-15Mo alloy and confirmed significant changes of elastic modulus. Zirconium, as an alloying element for titanium, is highly safe for the human body, as it is a non-toxic element, having shown no mutagenic or carcinogenic effects. An additional advantage is the fact that Ti--Zr alloys have very high corrosion resistance [7,8,9]. Tantalum is another biocompatible metal, due to its low toxicity and excellent corrosion resistance. Additionally, it has been shown that Ti-Ta alloys possess the lowest Young’s modulus values among materials currently used for implants. The Ta coat on a traditional titanium plate can promote fixing of bone fractures [10,11]. The studies of Zhou and Niinomi [12,13] have shown that Ti-Ta alloys produced by the arc melting method have strength in the range 510–690 MPa, depending on the percentage of tantalum. The second method for influencing the mechanical properties of the element—and, in particular, the value of the Young’s Modulus is to introduce porosity into the material. It is well-known that porosity has effects on mechanical properties, such as the strength or elasticity, of the whole element. Additionally, there have been reports focused on the effect of the porosity on the corrosion resistance of the material [14,15,16,17]. In the study of Oh et al. [18], a decrease of Young’s modulus with the introduction of porosity into the material was observed; moreover, a linear relationship between the porosity and elasticity was confirmed. According to the cited studies, a titanium-based alloy with a porosity of approx. 30% will have a modulus of elasticity similar to that of human cortical bone. Creating materials with a gradient structure of porosity for potential use as bone implants seems to be appropriate, due to the combination of functions performed by pores of specific size. Such a situation is attractive, due to the maximum strengthening of the bone–implant connection [19,20,21]. Additionally, the arrangement of pores of a particular size, and thus, the presence of areas of different porosity in a very specific way, can affect the mechanical properties of the whole element, as mentioned above. Recognition of the influence of particular areas will allow for determination of the possibility of designing implants with strictly defined mechanical properties individually adjusted to the bones of patients. The architecture of human bone itself shows heterogeneous porosity, which can be observed on the longitudinal section of long bones, consisting of both spongy and compact bone [22].

Functionally graded materials (FGMs) are materials whose structure, composition, or porosity is distributed in a gradient. The areas are characterized by microscopic areas of non-homogeneous material which, in the whole, form a gradient of the analyzed factor. For example, a dental implant material with a gradient composition ranging from pure titanium at one end to a mixture of titanium and 20% hydroxyapatite at the other end has been reported in the literature. Titanium, as a metallic element, is used to ensure the strength of the whole element, while hydroxyapatite has high biocompatibility with bone [23,24]. Torres et al. [22] have produced samples by mixing titanium powder with NaCl in different ratios and using different pressure during production. In addition to developing a procedure for the production of samples with a reproducible porosity structure, the authors used the ultrasound method to demonstrate its potential to improve the modulus of elasticity without a significant loss of strength. In subsequent studies, Torres et al. [25] produced samples with porosity varying along sample cylinders, using powder metallurgy and sintering. The porosity of the subsequent layers of material produced varied from 15-45%. The development of an appropriate porosity gradient improved the strength, compared to ordinary porous materials, and a change in the Young’s modulus, from 75 GPa to 34 GPa, was additionally noted.

The main aim of our research was to determine the possibility of producing Functionally Grade Materials comprised of three areas arranged concentrically, using powder metallurgy. The effects of the milling parameters on the degree of powder agglomeration and, consequently, the porosity of sintered samples were studied. 

## 2. Materials and Methods

**Figure 1:**
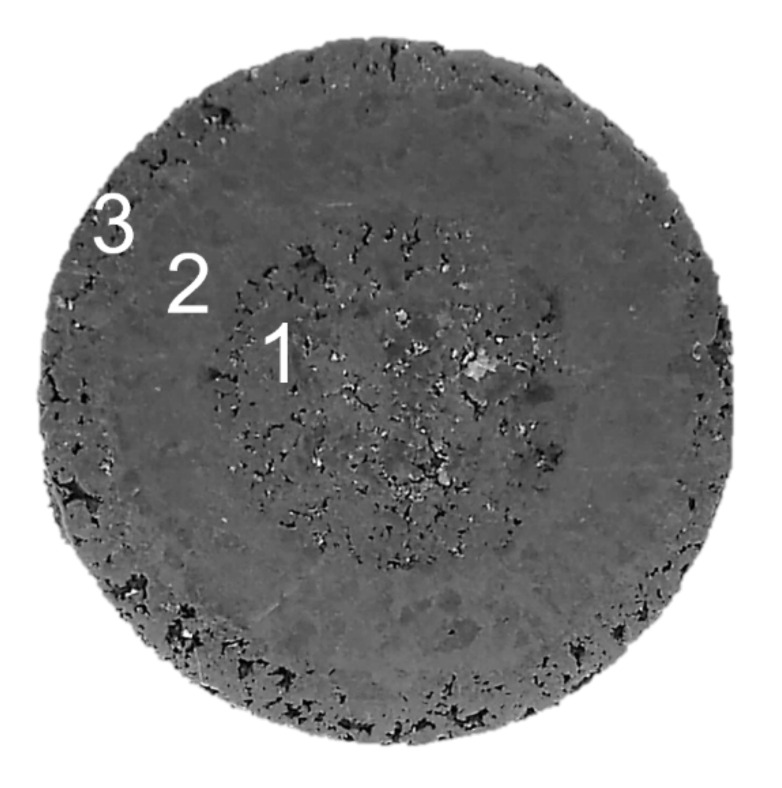
Schematic representation of sample construction.

Commercial powders of Ti (Atlantic Equipment Engineers (AEE); purity 99.5%,), Ta (Sigma Aldrich; 99.9%; 325 mesh; <44 μm), and Zr (Atlantic Equipment Engineers (AEE); purity 99.5%; particle size < 300 μm) were used for preparation of the initial materials. Zr elemental metal was used in commercial form. The titanium powder was agglomerated in a planetary-ball mill with containers and balls (10 mm) made from hardener steel, in order to obtain two different fractions, in terms of powder size (about 450 μm and 1000 μm). The process was performed by the following parameters: 200 rpm, charge weight 20 g, balls: powder ratio was 10:1 and process were performed 10/20 h. Additionally, they were blended Ti-50Ta (wt.%) and Ti-50Zr (wt.%) compositions. The process of mixing was performed by the following parameters: 100 rpm, charge weight 10 g, balls: powder ratio was 5:1 and process were performed 75 h. These two processes were performed in a planetary ball mill (Fritch Pulverisette 7 premium line) under a high-purity argon atmosphere (99.99%). The material was prepared without any substances to improve the porosity (e.g., space holders). The key task was the preparation of graded cylindrical green compacts comprised of three areas with different porosity and composition, as shown in Figure 1 and Table 1. This was achieved by three-step cold isostatic pressing. The samples were prepared without any binder or lubricant. Then, the green compacts were sintered in a vacuum at 1000 ℃ for 24 h and cooled in the furnace to room temperature. The sintered specimens for observation were ground and polished by a standard metallographic procedure and etched in Kroll’s solution.

The phase composition of the obtained material after sintering were studied by X-ray diffraction method (XRD), using a Phillips X-ray X’Pert diffractometer (PANalytical, Almelo, The Netherlands) with a lamp with a copper anode (CuKα-λ = 1.54178 Ǻ). The phase analyses were performed using the ICDD PDF-4+ database.

The microstructure of sintered materials was observed using the scanning electron microscope (SEM) JSM 6480 (JEOL, Tokyo, Japan) with an accelerating voltage of 20 kV. Additionally, we performed chemical composition analyses using an energy dispersive X-ray spectroscopy detector (EDS; IXRF, Austin, TX, USA) using the standard calibration method. 

The analysis of porosity in particular regions was performed by stereological methods based on optical microscope (OM; Olympus, Tokyo, Japan) imagery. A number of images were combined to obtain the largest possible area of analysis. The assessment of porosity and size of the pores was performed using the planimetric method with the ImageJ software (version 1.50b). We used the following stereological parameters: The surface area of the pores, a_p_ (μm^2^); volume fraction of the pores, V_V_ (%); the Feret diameter (largest distance between two points along the selection area); and the shape of the pores, which was assessed through the circularity parameter.

Micromechanical tests were performed using a Micro Combi Tester MCT3 (Anton Paar, Corcelles-Cormondrèche, Switzerland). Measurements were performed according to the recommendations of ISO 14577 [21]. A Berkovich indenter was used, with a maximum load 300 mN (0.3 N), loading and unloading time 30s each, and endurance time under maximum load of 10 s. Instrumental hardness (H_IT_) and instrumental elastic modulus (E_IT_) were determined by the Oliver–Pharr method [26]. Measurements were made to determine whether there was a gradual change in mechanical properties. Therefore, measurements were taken in a single line, moving from the center to the outside of the specimen.

The corrosion resistance measurements were conducted in a 250 mL glass cell with temperature control. The samples were mounted in resin with a copper wire, and their surface was ground to a surface finish of P4000. The resulting samples had a geometrical surface area of 1.131 cm^2^. Apart from the sample (serving as the working electrode), two more electrodes were inserted into the cell. The reference electrode for the experiments was a saturated calomel electrode (SCE), while the counter-electrode was a platinum mesh. The studies were performed in naturally aerated Ringer’s solution (8.6 g/dm^3^ NaCl, 0.3 g/dm^3^ KCl, and 0.48 g/dm^3^ CaCl2·6H_2_O; Fresenius Kabi, Poland) at 37 °C. The measurements were controlled using a potentiostat–galvanostat (PARSTAT4000A, Ametek, Princeton Applied Research/Solartron, Tennessee, USA/the U.K.) with the VersaStudio software. The following experimental procedures were performed: open-circuit potential (EOCP) stabilization for 7200 s; and potentiodynamic polarization (PDP), starting from −200 mV vs. EOC up to 1500 mV vs. SCE with a scan rate of 20 mV/min. When the current exceeded 50 μA/cm^2^, the polarization direction was reversed, in order to check whether the rise in the current was due to the breakdown of the passive oxide layer on the substrate.

## 3. Results and Discussion

**Figure 2:**
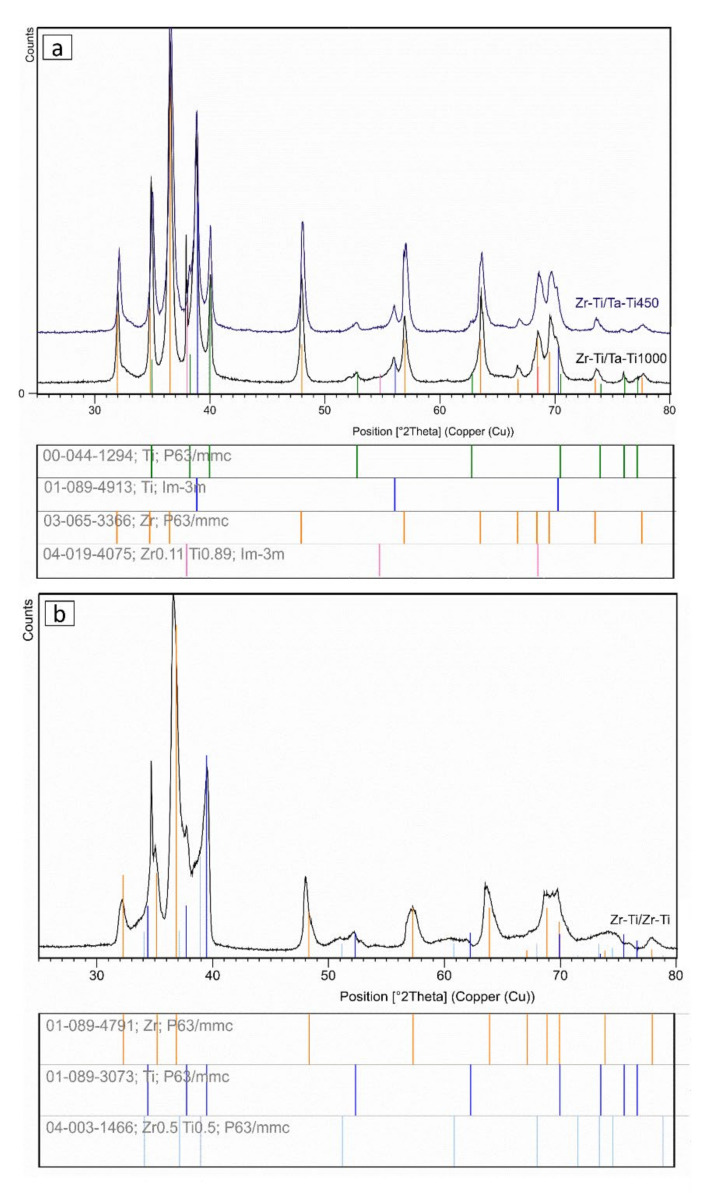
X-ray diffraction patterns of: (**a**) Zr–Ti/Ta–Ti system; and (**b**) Zr–Ti/Zr–Ti system.

The XRD diffraction patterns of the sintered samples revealed the presence of four different phases. For Zr-Ti/Ta-Ti samples (see Figure 2a), we observed the following phases: α-Ti (ICDD PDF 01-089-4913), β-Ti (ICDD PDF 00-044-1294), Zr (ICDD PDF 03-065-3366), and Zr0.11Ti0.89 (ICDD PDF 04-019-4075). The α-Ti and Zr phases were consistent with the initial materials, while the appearance of β-Ti was related to the presence of tantalum in the ring composition. The Ti-50Ta wt.% composition was pre-mixed, but sintering resulted in synthesis, indicating the presence of β-Ti. Pure tantalum is a good β-stabilizing element of titanium. The literature has indicated that as little as 25% wt. Ta can cause the appearance of the β-phase and, with increasing tantalum content, the β-phase proportion increases [13,27,28,29,30]. We also detected a non-stoichiometric phase of Zr0.11Ti0.89, likely located in the transition zones. In the samples, the zirconium and pure titanium zones had no mechanical contact. However, we suppose that diffusion allowed for the formation of a non-stoichiometric phase. It should be noted that the contact area between titanium and zirconium in the gradient material was found to be relatively small, compared to that in the conventional blending of powders. 

In case of Zr-Ti/Zr-Ti samples (Figure 2b), we found three phases. Two of them, α-Ti (01-089-4913) and Zr (03-065-3366), were similar to that of the initial material. Additionally, a Zr0.5Ti0.5 (04-003-1466) phase was present. The direct contact between Ti and Ti-50Zr, as well as Zr and Ti-50Zr, allowed for the diffusion. Generally, diffusion proceeds in the direction of the chemical potential gradient, resulting in the formation of a stoichiometric phase. The Ti-Zr phase diagram revealed the complete solution over the whole range of components. The interdiffusion in a binary solid system is determined by the flux of the atoms diffusing in the direction of the chemical potential gradient. For the Ti-Zr binary system, interdiffusion is dominated by the vacancy mechanism of diffusion and determined by the diffusion coefficients of the titanium and zirconium atoms [31,32,33,34].

Observation of all samples at lower magnification demonstrated differences between samples with transition zone between Ti-50Ta wt.% and Ti-50Zr wt.%. In the samples containing tantalum, more distinct transition zones could be distinguished. Additionally, observation revealed, for all samples, a continuous and gradual change in microstructure. However, it is clear that all the samples had three areas.

**Figure 3:**
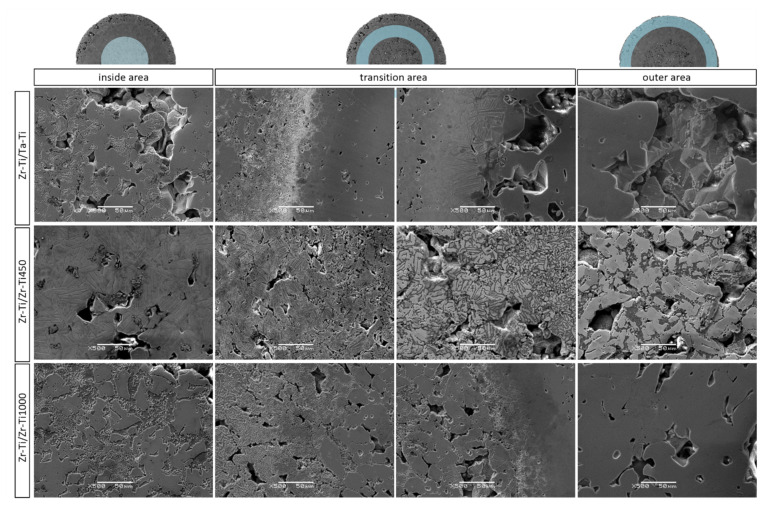
SEM images, with higher magnification, of the sintered samples.

Observations at higher magnification indicated that the microstructure changed in a manner dependent on the distance from center of the sample (Figure 3). For all samples, the center was zircon with the same gradation and, therefore, for all samples, this part revealed a microstructure characteristic of zircon, with similar pore structure. The transition zone in all samples revealed a transitional and continuous change in the microstructure. Additionally, for all cases, a significant microstructure was observed in this zone, with needle-shaped grains. The outer part was formed of titanium after agglomeration, as confirmed by observation of the microstructure, which was characteristic of titanium. More importantly, for all cases, there were solid connections between the powder particles, regardless of the position in the sample. The sintering and diffusion processes that create these solid interfaces allow for the formation of permanent bonds between the different areas, which is crucial in the case of samples comprised of different zones. 

**Figure 4:**
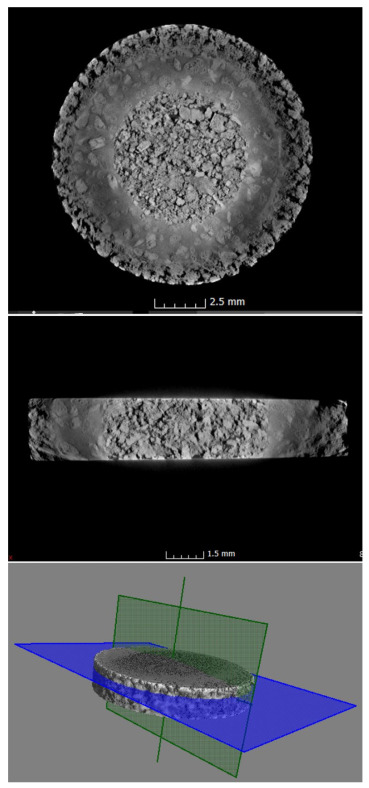
Microtomography observations of the Zr-Ti/Zr-Ti sample. Cross-section of the specimen (top photo), section along the specimen (middle photo) and in the last photo the location where the above images were taken.

In order to confirm the repeatability of this structure in the whole volume of the samples, observations of the 3D structure were carried out through the use of X-ray tomography (see Figure 4). We confirmed that all of the samples preserved the three-zone structure in their whole volume. Additionally, no anomalies were observed in the construction of the whole sample, indicating that three-stage isostatic pressing allows for reproducible construction. We only observed differences in the case of the ring; however, this was due to the fact that the ring itself was made of fine powder after mixing, and this powder filled the empty spaces in the inner and outer zones to a varying extent. 

**Figure 5:**
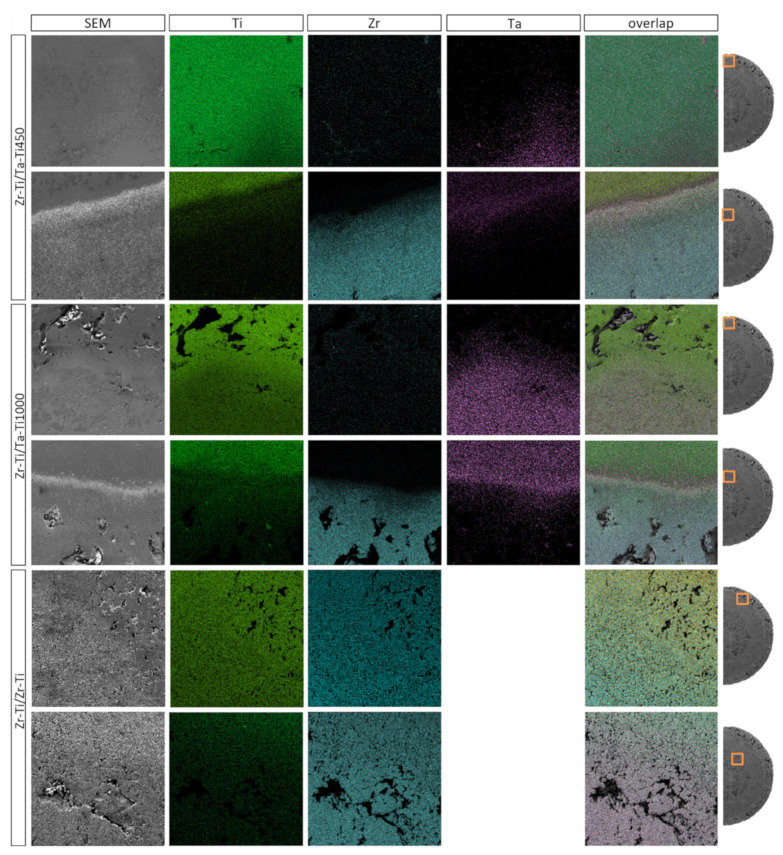
SEM images and distribution elements maps for all samples, with marked observation locations.

**Figure 6:**
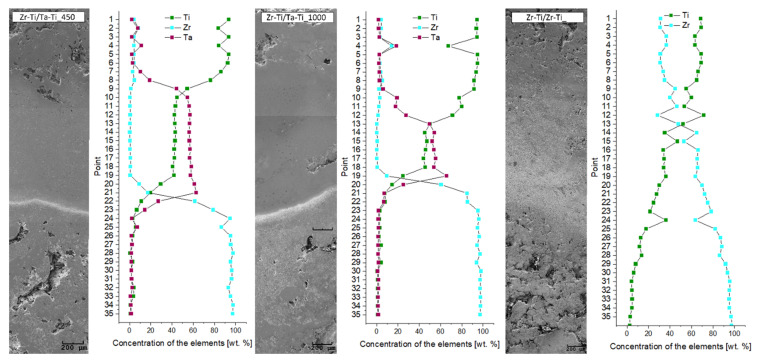
Quantitative analysis of chemical composition, in relation to the location at which the measurement was made. Point 1 is for the outside sample edge and with increase of number the measurement it is closer to zirconium core (point 35).

The elemental distribution maps (see Figure 5) in the transition zones between the core and ring and between the ring and outer zone clearly illustrate the degree of elemental diffusion between the particular zones. These observations were also complemented by a quantitative analysis of the concentration of individual elements as a function of distance from the sample center, as shown in Figure 6. It is worth noting that quantitative chemical composition measurements were conducted at equal intervals, in order to ensure the most meaningful results across the sample cross-section.

For samples with a ring having a Ti-50Ta wt.% composition, we observed a smooth transition of tantalum concentration towards the outer layer. Moreover, in the outer layer, we observed small amounts of zirconium, which may indicate that there was a diffusion process of the zirconium, from the center of the sample to the outer zone. In the case of the core–ring diffusion zone, we observed a more edgy cut-off between the components; which was very well observed for zirconium and tantalum. In contrast, a much better diffusion of titanium towards the zirconium core was observed. The different diffusion coefficients of Ti and Ta in the structure resulted in the unbalanced transport of atoms from one zone to the other [35,36]. Ansel et al. [36] have shown that the diffusion coefficient in Ti-Ta alloys strongly depends on the concentrations of the elements. Thus, at a lower tantalum content, this coefficient is many times higher than for a higher tantalum concentration. Therefore, it is reasonable to consider rings with lower tantalum content; in particular, considering that a much lower content of tantalum (approx. 25%) can allow for the appearance and stabilization of a β-titanium phase [27]. It should be mentioned that diffusion is limited in porous materials, where diffusion of the elements takes place within the diffusive necks between the powder particles. 

Quantitative analysis of pores revealed differences between the samples, due to the different porosity of the outer zone. For the sample in which a fraction of smaller titanium particles was used for the outer layer, a significant elongation of the ring–external diffusion zone was observed. This is likely related to the fact that the smaller particles of powder effectively obtained a smaller porosity and, thus, there was more material at the interface between the two areas, which allowed for more efficient diffusion of tantalum. It is worth noting that the use of a fine powder after mixing to create a ring allowed for a better connection with more porous materials, both inside and outside. In addition, unfortunately, the zirconium diffusion region towards the Ti-50Ta wt.% ring was found to be small. We can clearly see from the graphs that the change in zircon concentration was rapid. In contrast, in the case of the Zr-Ti/Zr-Ti structure, we observed a gradual and gentle change of the components. Introducing a Ti-50Zr wt.% ring affected a slight change in the concentration of the components, additionally extending the zone of occurrence of diffusion. Quantitative analysis showed that approximately 20% of zirconium was observed in the outer zone of the sample and a low percentage of titanium was observed in the center of the sample. In this case, the diffusion of the components took place over the entire cross-section of the sample. Titanium and zirconium have the same crystal structure and belong to the same group in the periodic table of elements, which greatly promotes their interdiffusion. The phase diagram revealed the complete solution over the whole range of components [33,34]. Additionally, the Ti-50Zr wt.% composition material showed low activation energies, similar to the values previously reported for pure Zr or Ti [31]. 

The presence of pores in materials for potential use as long-lasting implants can decrease the stiffness of whole element. The percentage of the pores (volume fraction) in the observation area for particular areas was estimated, based on the pore surface fraction on the material assessed using optical microscope (Table 2). For the inside zone, composed of the same material, similar levels of porosity were observed for all three sample types, between 20 and 30%. Closer analysis of the cross-sectional area revealed the first differences for the inner part of the sample. In the Zr-Ti/Ta-Ti450 sample, more than 22% of the pores were larger than 500 μm^2^. In the case of the Zr-Ti/Ta-Ti1000 sample, the amount of these pores was 7% and, for the Zr-Ti/Zr-Ti sample, it was 25%. Differences in porosity appeared at the ring zone for samples where the transition zone was a mixture of Ti-50Ta wt.%. For the Zr-Ti/Ta-Ti450 sample, the porosity of this region was 7% while, for the Zr-Ti/Ta-Ti1000 sample, it was 6%. In contrast, for the sample in which the transition zone was a mixture of Ti-50Zr wt.%, the volume fraction of pores was 12%. It is worth noting that the size of the powders was not observed after mixing, regardless of the composition; therefore, this difference in porosity was not due to the particle size. It can be assumed that diffusion is important here, which occurs much more easily for binary systems, in terms of the whole sample, than when considering three components. 

The specimens were designed, based on literature reports, to support osteointegration as much as possible and, thus, to improve the implant–bone interface. The volume fraction of pores in the outer zone was 44% for the Zr-Ti/Ta-Ti450 and Zr-Ti/Zr-Ti samples. In both of these cases, the outer layer was composed of particles that were homogenized in a uniform manner, suggesting that the porosity results are reproducible. The presence of interconnected pores may allow for the entry and flow of body fluids and nutrients, which is why the presence of pores with a large cross-section is so important. When it comes to the outer layer, designed for bone contact, not only is the overall porosity important, but also the structure and shape of these pores. This is extremely important for osteointegration. Research in the literature [19,37,38] has demonstrated that a pore size (Feret diameter) greater than 100 μm is suitable for cell migration. The presence of larger pore sizes supports further important processes, such as osteoprogenitor cell migration, adhesion, and proliferation of cells. The average pore cross-section sizes understood as cross-section area for Zr-Ti/Ta-Ti450, Zr-Ti/Ta-Ti1000, and Zr-Ti/Zr-Ti samples were 4752 μm^2^, 1488 μm^2^, and 3670 μm^2^, respectively. In general, for the outer layer, we noted differences in the number of pores with cross-section area larger than 500 μm^2^. The Zr-Ti/Ta-Ti450 sample showed more than 28% of these pores. In the case of the Zr-Ti/Ta-Ti1000 sample, the amount of these pores was 14% in the outer region while, for the Zr-Ti/Zr-Ti sample, it was 10%. The microscopic observations of the microstructure and the results of the analysis of the presence of pores with such a large cross-section suggest the presence of interconnected pore systems. This is extremely important, in terms of facilitating the penetration of body fluids and nutrients to ensure cell nourishment [21]. Another stereological quantity analyzed was Feret diameter, understood as the distance between the two parallel planes restricting the object perpendicular to that direction. The maximum Feret diameters for Zr-Ti/Ta-Ti450, Zr-Ti/Ta-Ti1000, and Zr-Ti/Zr-Ti samples were 2323 μm, 2158 μm, and 1510 μm, respectively. As above, the percentage of pores for which the Feret diameter was greater than 100 um was performed. The Zr-Ti/Ta-Ti450 sample showed more than 21% of these pores, the Zr-Ti/Ta-Ti1000 samples had over 12% in the outer region and, for the Zr-Ti/Zr-Ti sample, it was 10%. The dimensionless coefficient of circularity for the outer layer of all sample types was 0.5. Contrary to predictions, the Zr-Ti/Ta-Ti1000 sample-that is, the one where larger particles were used for the outer layer—revealed a smaller proportion of large pores in both the inner and outer regions. Previous studies have revealed that the larger the powder particles used, the greater the porosity obtained and the proportion of pores with a large cross-sectional area. However, in the case of gradient materials, when we used a material with large particles for the outer layer, these values changed. This may be related to the three-step isostatic pressing process, having a relatively small space for the outer layer. This led to a tighter compression of both the outer and inner layers, through compression from the outside. In addition, the agglomeration of powders of different sizes may have resulted in powders of different sizes being in different locations between the curing and plasticizing of the material. In powder metallurgy, these processes follow one another cyclically; which is evident from the theory of the method, as well as from previous studies.

**Figure 7:**
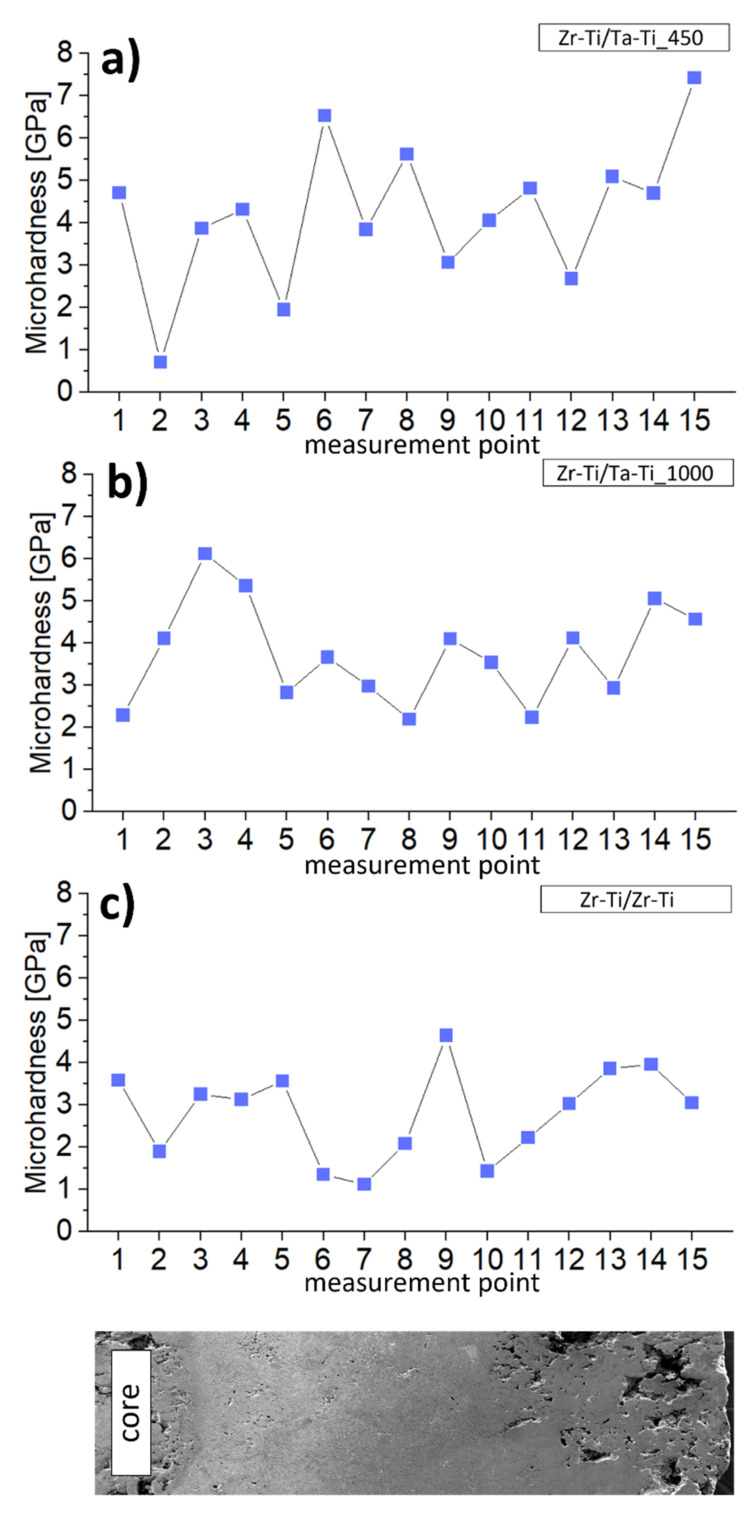
The relationship between the microhardness and measurement location (from core to outside zone of samples, representation at the bottom) for samples (**a**) Zr-Ti/Ta-Ti450, (**b**) Zr-Ti/Ta-Ti1000, (**c**) Zr-Ti/Zr-Ti.

**Figure 8:**
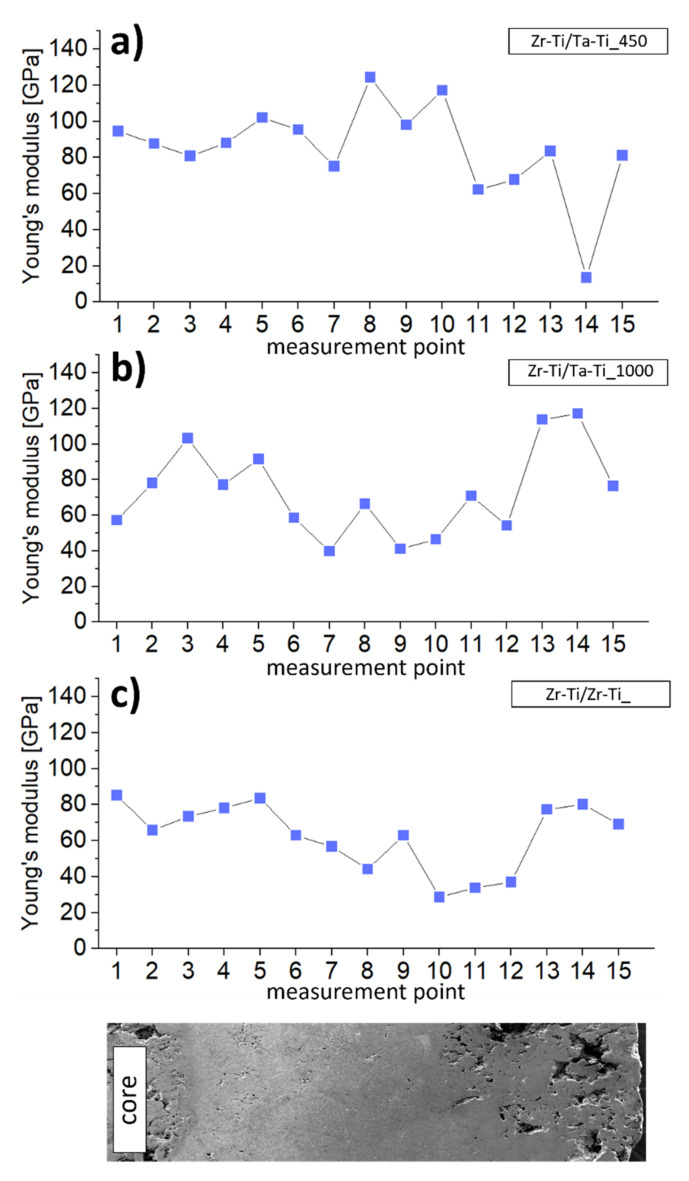
The relationship between the Young’s modulus and measurement location (from core to outside zone of sample, representation at the bottom) for samples (**a**) Zr-Ti/Ta-Ti450, (**b**) Zr-Ti/Ta-Ti1000, (**c**) Zr-Ti/Zr-Ti.

In an attempt to examine the mechanical properties of the samples, we performed microhardness and instrumental Young’s modulus measurements across the samples (Figure 7 and Figure 8). Points 1–3 were for the zirconium core, while points 4–10 were for the Ti-50Zr or Ti-50Ta wt.% ring and the remaining points were for the outside area. For all samples, we observed gradual changes of analyzed mechanical parameters, particularly in terms of the transition zones between the areas. It is worth noting that this change was generally not continuous over the entire sample. First, this may have been related to the different degree of diffusion of components from the ring towards the core. The second reason for these differences may be the high porosity of the core. For Zr-Ti/Ta-Ti samples, areas of increased microhardness were observed around the 3rd or 4th measurement point, while the same was observed for the Zr-Ti/Zr-Ti sample around the 9th measurement point. These areas coincide with the significantly smaller needle-like grains in the transition zones in the aforementioned samples, which were related to a local increase in microhardness. The lowest microhardness values were found throughout the Zr-Ti/Zr-Ti sample section. When measuring Young’s modulus, we observed much smaller changes in values within samples. Additionally, measurements in relation to the position on the cross-section of the specimen perfectly illustrated the continuous change of values, with particular emphasis on the transition areas. More importantly, for many points in the Zr-Ti/Ta-Ti1000 sample and for almost the entire Zr-Ti/Zr-Ti sample, the Young’s modulus value was below 100 GPa. This is very important for the potential application of the designed materials in long-term bone implants. It has been estimated that the value of Young’s modulus of bone varies from 10 to 40 GPa, depending on the bone tested; however, the Young’s modulus of pure titanium (αTi) is 105 GPa, while that for the most commonly used alloy in medicine, Ti-6Al-4V, is 110 GPa [39,40,41].

**Figure 9:**
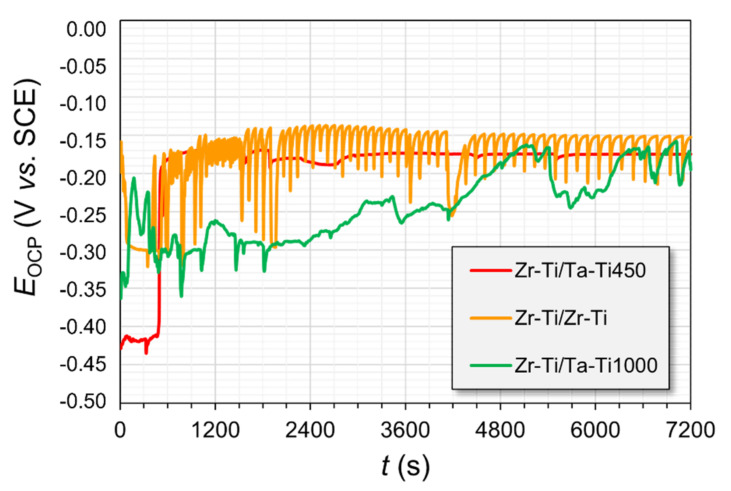
Open-circuit potentials (EOCP) registered for the metal samples during immersion in Ringer’s solution.

The samples that were subjected to the corrosion studies were first mounted in a resin, which was utilized to limit the surface area of the cross-section of the material with different core-shell configurations of microstructure, as well as composition. The porous subsection of the materials was prone to becoming infiltrated by the corrosion medium during the measurements, which was readily visible in the case of the E¬OCP time progression curves (see Figure 9). The effect was the most pronounced for the group of materials that had a pure zirconium phase embedded within their architecture.

**Figure 10:**
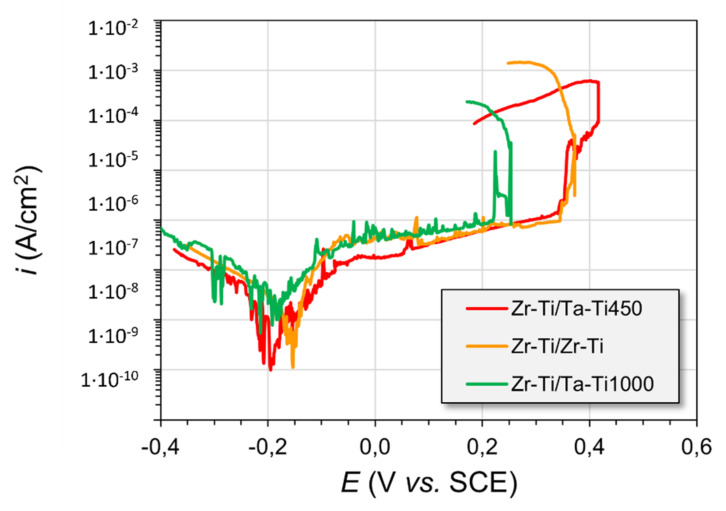
Potentiodynamic polarization (PDP) curves, recorded for the metal samples after EOCP stabilization in Ringer’s solution.

For these specimens, there were quite significant oscillations in the measured potential throughout the experiments. The Zr-Ti/Zr-Ti sample experienced the most pronounced changes. The reason for this observation might be the slow infiltration of the electrolyte through the intricate pore architecture of the material. The fact that the material had different metallic phases interlocked into a single sample gave rise to the possibility of galvanic coupling between the less noble (anodic) and more noble (cathodic) regions. As a result, the formation of corrosion products could obstruct the channels within the material, thus limiting the continued ingress of Ringer’s solution. From the point of view of the possibility of ingrowth of body tissue within the material, the results suggesting the continual wetting of the inner surfaces of the immersed samples indicate some promise for their potential use in bone reconstruction. The potentials of the Zr-rich samples were characterized by similar E_OCP_ (open-circuit potentials) values at the end of the stabilization period (Table 3), which were around −175 mV vs. SCE. The corrosion potential of pure zirconium in Ringer’s solution is typically in the range of −400 to −350 mV vs. SCE [34,42]. The PDP results signified that almost all of the studied materials had undergone passive oxide film breakdown during the polarization scans (Figure 10). The rupture of the protective layer may give rise to a lack of integration of the tissue with the medical device or lead, in extreme cases, to the failure of the implant as a result of corrosion and metallosis. The corrosion potentials (E_cor_) of the samples, as measured from the PDP curves (Figure 9 and Table 3), were slightly shifted towards more negative potentials (as compared with the stable E_OCP_ values), due to the nature of the scan. The sweeping of potential generates a capacitive charging current through the electrochemical interface, which skews the current readings to higher values (and the E @ i = 0 point to more negative potentials). The E_bd_ potentials were increasing in the order Zr-Ti/Ta-Ti1000 < Zr-Ti/Ta-Ti450 < Zr-Ti/Zr-Ti; however, these results may be inconclusive, due to the fairly wide range of the measured data.

## 4. Conclusions

For this paper, a powder metallurgy method was successfully used to produce materials with gradient composition and structure (with reference to porosity) in the whole element. The materials were obtained from powders, which were properly prepared and subjected to three-step isostatic pressing. The extensive characterization of the materials allowed us to draw the following conclusions:

Microscopic observation confirmed a gradual change in microstructure, depending on the distance from the center of the sample. In addition, chemical composition analysis confirmed the occurrence of diffusion processes, despite the high porosity of the outer and inner zones. Qualitative analysis by X-ray diffraction also confirmed these results. 

Stereological analysis demonstrated the variation of porosity in individual zones. In addition, the porosity of the inner and outer layers was quite significant, which can effectively affect the properties of the whole component. In addition, the pore structures partially complied with the requirements listed in the literature as being optimal for osteointegration.

The mechanical parameters were found to be differentiated through the studied areas of the samples, with a clear increase in the parameters within the transition zones between the individual zones. 

Corrosion tests were performed, confirming the suitability of the developed graded materials for application in moderately oxidizing environments. From the point of view of the possibility of ingrowth of body tissue within the material, results suggesting the continual wetting of the inner surfaces of the immersed samples show promise for their potential use in bone reconstruction.

## Figures and Tables

**Table 1:** Schematic representation of sample construction.

Sample	Zr-Ti/Ta-Ti450	Zr-Ti/Ta-Ti1000	Zr-Ti/Zr-Ti
Area1-inside	Zr	Zr	Zr
Area2-ring	Ti-50Ta	Ti-50Ta	Ti-50Zr
Area3-outer	Ti-450	Ti-1000	Ti-1000

Area1/2/3 is mark in Figure 1.

**Table 2:** The volume fraction of particular areas of all samples.

	Zr-Ti/Ta-Ti450	Zr-Ti/Ta-Ti1000	Zr-Ti/Zr-Ti
Area 1-inside	21%	25%	29%
Area 2-transition	7%	6%	12%
Area 3-outer	44%	25%	44%

**Table 3:** Electrochemical corrosion investigations results. All potentials are reported with respect to SCE.

Sample	E_OCP_ (mV)	E_cor_ (mV)	E_bd_ (mV)
Zr-Ti/Ta-Ti450	−174 ± 22	−194 ± 23	357 ± 5
Zr-Ti/Ta-Ti1000	−195 ± 81	−220 ± 43	309 ± 46
Zr-Ti/Zr-Ti	−152 ± 13	−175 ± 4	428 ± 86

## Data Availability

Not applicable.
